# A preliminary comparison of a songbird’s song repertoire size and other song measures between an urban and a rural site

**DOI:** 10.1002/ece3.8602

**Published:** 2022-02-16

**Authors:** Dustin E. Brewer, Adam M. Fudickar

**Affiliations:** ^1^ Environmental Resilience Institute Indiana University Bloomington Indiana USA; ^2^ Present address: 5649 Central Michigan University Mount Pleasant Michigan USA

**Keywords:** note rate, nutritional stress hypothesis, peak frequency, song complexity, song sparrow, urbanization

## Abstract

Characteristics of birdsong, especially minimum frequency, have been shown to vary for some species between urban and rural populations and along urban–rural gradients. However, few urban–rural comparisons of song complexity—and none that we know of based on the number of distinct song types in repertoires—have occurred. Given the potential ability of song repertoire size to indicate bird condition, we primarily sought to determine if number of distinct song types displayed by Song Sparrows (*Melospiza melodia*) varied between an urban and a rural site. We determined song repertoire size of 24 individuals; 12 were at an urban (‘human‐dominated’) site and 12 were at a rural (‘agricultural’) site. Then, we compared song repertoire size, note rate, and peak frequency between these sites. Song repertoire size and note rate did not vary between our human‐dominated and agricultural sites. Peak frequency was greater at the agricultural site. Our finding that peak frequency was higher at the agricultural site compared to the human‐dominated site, contrary to many previous findings pertaining to frequency shifts in songbirds, warrants further investigation. Results of our pilot study suggest that song complexity may be less affected by anthropogenic factors in Song Sparrows than are frequency characteristics. Additional study, however, will be required to identify particular causal factors related to the trends that we report and to replicate, ideally via multiple urban–rural pairings, so that broader generalization is possible.

## Graphical abstract

Urban factors are known to influence the health of birds, which could be indicated by vocal signals like song type repertoire size. We compared song type repertoire size, and other characteristics related to song, between an urban and rural site in Song Sparrows (Melospiza melodia). Our results suggest that song type repertoire size was not affected by urban factors, though urban birds tended to sing songs with a lower peak frequency.

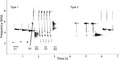

## 1 INTRODUCTION

Urban land cover is currently between 2% and 3% of total global land area when Antarctica and Greenland are excluded (Liu et al., 2014). Between 1970 and 2000, urban land cover increased globally by about 58,000 km^2^ and will likely increase by at least an additional 1,000,000 km^2^ between 2000 and 2030 (Seto et al., 2011). This will constitute about a 33% increase in urban land cover. Urbanization introduces novel challenges to wildlife, including artificial light at night, noise pollution, and modification of habitat structure (Shanahan et al., 2013). As global urban land cover rapidly increases, it is becoming increasingly important to understand behaviors of animals that live in urbanized habitats, especially in comparison with conspecifics at non‐urbanized sites. Such comparisons can identify ways that species that occur in urban areas are affected by urbanization (Tuomainen & Candolin, 2011), and ultimately could help to design cities to minimize negative impacts upon urban wildlife.

Birdsong is known to function both in mate attraction and territory defense (Catchpole & Slater, 2008). Because this behavior is conspicuous, it can, with relative ease, be compared between urban and rural environments to better understand how urbanization affects information signaling. For example, songs of Northern Cardinals (*Cardinalis cardinalis*) have been shown to advertise territory quality at rural sites but not at urban sites (Narango & Rodewald, 2017). The minimum frequency in birdsong has been shown in some species to be higher at noisier sites (Seger‐Fullam et al., 2011; Slabbekoorn & Boer‐Visser, 2006), probably to focus the vocal signal above low‐frequency urban noise. Such noise may compromise the ability of males to attract females who prefer (a) lower frequency songs (Huet des Aunay et al., 2014) and/or (b) ‘high performance’ songs, which maximize both trill rate and frequency bandwidth (Luther et al., 2016).

In addition to frequency and ‘performance’ characteristics, song complexity can also indicate the quality of singing males (e.g., Boogert et al., 2008). For an individual, song complexity can be measured at the within‐song level by computing the total number of notes or note types, syllables or syllable types, and phrases or phrase types. A note is a continuous trace on a spectrogram, a syllable is a series of notes always uttered together, and a phrase is a series of syllables always uttered together (Baker, 2001). More phrases (Leitão et al., 2006) and total number of notes (Wasserman & Cigliano, 1991) per song have been shown to elicit more responses from captive female songbirds and, in the field, males with more syllables in their songs have had mates which initiated egg‐laying earlier (Mennill et al., 2006). Similarly, the rate at which such song elements are uttered within songs, even without respect to bandwidth (i.e., ‘performance’), could feasibly indicate a ‘temporal complexity’ that could also be important for signaling to conspecifics. This type of within‐song complexity has been shown to be decreased by urban factors in multiple species (Hill et al., 2018; Potvin et al., 2011). Previous investigators have sometimes not referred to note—or other song element—rates as ‘complexity,’ though we do herein because the presence of more elements per unit time is more complex per se.

Song complexity can also be measured at the between‐song level by counting the same units overviewed above (i.e., distinct notes, syllables, and/or phrases) throughout a repertoire or by counting the number of distinct song types displayed as defined by these components (e.g., MacDougall‐Shackleton et al., 2009 used both approaches). This variability can be informative to conspecifics. For example, field studies have shown that female Song Sparrows (*Melospiza melodia*) likely prefer males that sing more song types (e.g., Reid et al., 2004). Studies have also shown that anthropogenic noise is negatively correlated with repertoire‐wide song complexity (e.g., via song elements; Juárez et al., 2021) and that urban noise exposure during nestling development is associated with smaller brain regions linked to song learning (Potvin et al., 2016). However, no study that we are aware of has compared the number of song types in song repertoires (hereafter ‘song repertoire size’) between an urban and rural environment.

Comparison of song repertoires between urban and rural sites could feasibly be used to evaluate the effects of urbanization on bird condition. There is evidence that song repertoire size can be an honest signal of male quality due to the nutritional requirements for song development (Nowicki et al., 1998, 2002). In Great Reed Warblers (*Acrocephalus arundinaceus*), for example, inner primary feather length of nestlings, a proxy for condition, was positively correlated with subsequent song repertoire size (Nowicki et al., 2000). Different studies have found different results regarding avian body condition between urban and rural sites (urban birds in worse condition: Heiss et al., 2009; Liker et al., 2008, urban birds in better condition: Santiago‐Alarcon et al., 2018). Urban settings could increase or decrease food availability, lengthen photoperiod, and introduce noise such that body condition is affected. For example, urban noise can reduce parental provisioning rates which likely affects offspring condition (Lucass et al., 2016). Variance in mean song repertoire size between urban and rural sites could feasibly offer a less invasive way than catching and handling birds to evaluate the effects of urbanization on bird condition.

The Song Sparrow is ideal for comparing urban and rural sites with respect to song repertoire size because this species is commonly found in both urban and rural habitats throughout much of North America. Further, Song Sparrows have been studied extensively with respect to singing behavior (e.g., Hiebert et al., 1989; Reid et al., 2004; Searcy et al., 1995, and many others), which is helpful both for methodological and comparative purposes. Song repertoires displayed by Song Sparrows are crystallized after an individual's first spring (Nordby et al., 2002), which minimizes age effects when comparing between individuals. Importantly, Song Sparrows sing repertoires of about 4 to 13 distinct song types, as well as complex songs with many note and syllable types, which display sufficient compositional and temporal variability to correlate with possible effects of urbanization.

We compared Song Sparrow song between an urban, ‘human‐dominated’ site (where human occurrence was frequent) and a rural, ‘agricultural’ site (where human occurrence was rare). Our primary variables of interest for this pilot study were song repertoire size and note rate, which were used to describe song complexity. We predicted that mean song repertoire size at the human‐dominated site would be smaller than at the agricultural site due to increased anthropogenic disturbance at the human‐dominated site. For the same reason, we predicted that individual songs from the human‐dominated site would be less temporally complex (fewer notes per second) compared to songs from the agricultural site. We also compared peak frequency between the sites in order to determine if birds at our human‐dominated site were experiencing selection similar to other urban populations, where peak frequency has shifted upward, probably in response to low‐frequency noise (e.g., Walters et al., 2019). We predicted that noise would mostly affect song frequency characteristics, rather than habitat structure which could result in opposite effects (Job et al., 2016), and so that peak frequency would be higher at the human‐dominated site.

Our goal was not to draw general conclusions about the effects of urbanization and/or nutritional stress on the song characteristics that we measured. Rather, we sought to provide results of a simple comparison of song characteristics between an urban and a rural site. Our goal was to provide preliminary results to investigators interested in exploring in more depth how song characteristics, especially song complexity, relate to urban factors and/or to the condition of individual birds.

## 2 METHODS

### 2.1 Study sites

Our field sites were located in Indiana, at 39.17°N, 86.53°W. Field work occurred between April and July in 2018 and 2019. The agricultural site was located 6 km east of the city limits of Bloomington, which is well beyond the distance that young Song Sparrows likely disperse (Arcese et al., 2002). Fieldwork at our agricultural site was conducted on state‐owned land that was managed for wildlife, but which was leased to farmers. Fallow fields dominated this study site, though there were also portions covered by corn and soybeans. Song Sparrows occurred at the edge between fields and moderately sized (<50 m wide) bands of riparian forest dominated by silver maple (*Acer saccharinum*) that bordered Salt Creek and Brummett's Creek. Both creeks flooded during the spring (both years), covering the surrounding fields and likely affecting use by Song Sparrows. Aside from the activity of planting and harvesting crops, little human presence occurred at this site. We recorded Song Sparrows at our agricultural site within a 2‐km‐diameter area. The human‐dominated site was located on the campus of Indiana University, in Bloomington. Though the campus was covered by a relatively large area of green space, it was typical of many university campuses in that there were many sources of noise and artificial light at night, as well as a large proportion of area covered by impervious surface and regularly mowed areas. Humans generally occurred many times each day in Song Sparrow territories at this site. Most Song Sparrow territories on campus were centered along narrow (<10 m) riparian strips bordering Clear Creek or its tributaries, though some territories occurred adjacent to buildings where ornamental shrubs, primarily, provided cover. All of the Song Sparrows that we recorded on campus occurred within a 1.25‐km‐diameter area. We compared noise levels and impervious surface coverage between the human‐dominated and agricultural sites to confirm that the sites did vary regarding factors associated with urban impact.

We measured noise levels between 8:30 and 9:30 a.m. (morning session) and between 12:30 and 1:30 p.m. (afternoon session) in the center of five randomly selected Song Sparrow territories at both the agricultural and human‐dominated site. The same points were sampled during both the morning and afternoon at both sites on two different days, at least 4 days apart, when wind speeds were less than 25 km/h. Measurements were made using a ‘Radio Shack, 33–3042’ Super‐Cardioid Dynamic Microphone that was attached to a tripod, so that the top of the microphone was 1 m above the ground. The microphone was connected to a Tascam DR 100MKIII Linear PCM Recorder. For each recording, the gain of the recorder was set to the maximum (‘56.5’), the sample rate was 48 kHz, and the bit rate was 1152 kbps. We calculated ‘average power’ (dB) during a 2‐min period for each recording session by using Raven Pro 1.5 (Bioacoustics Research Program, 2019). Settings in Raven were the default (window type = Hann, FFT window size = 512). Average power values were measured between 0 and 10 kHz to calculate a mean value for each 2‐min period at each point. These mean average power values for all points measured at a given site during the morning session were averaged across both days. The afternoon session was treated the same. The dB values that we report are relative to each other and so effectively quantify noise amplitude between our sites, though do not represent absolute sound levels that a sound level meter would have generated and so should not be compared to such measures.

In the center of five randomly selected territories at both the human‐dominated and agricultural sites, we manually measured proportion of impervious surface within a 50‐m radius of each territory center using ArcGIS 10.4.1. Territories were defined by the area that birds were observed using and defending during recording sessions.

### 2.2 Song recording and analysis

We recorded entire song repertoires of territorial male Song Sparrows. By April, we assumed that second year birds had already acquired a crystallized song repertoire (Nordby et al., 2002). Individuals were randomly selected with the constraint that birds could only be considered for selection if they were singing at least five times per min on average, which was dependent upon breeding stage (e.g., nest building and egg laying). At both sites, apparently unpaired birds (5 of 12 in the human‐dominated site; 6 of 12 in the agricultural site) were recorded in addition to paired birds. Recordings were made throughout the day, but typically between 6 a.m. and 11 a.m. All but two of the Song Sparrows were color banded when they were recorded which aided in ensuring that we recorded exclusively the focal bird. The two individuals that were not color banded were carefully observed during the entire recording session, during which we were particularly conservative about deciding when to record the bird (i.e., only when it was occupying central parts of its territory). We used a Tascam recorder (DR‐100MKIII Linear PCM Recorder), which produced .wav files at a sample rate of 48 kHz and a bit rate of 1152 kbps. A shotgun microphone (Audio‐Technica AT8035) was used for recordings. We stood approximately 10 m from focal birds when recording. Playback was not used to induce singing.

**FIGURE 1:**
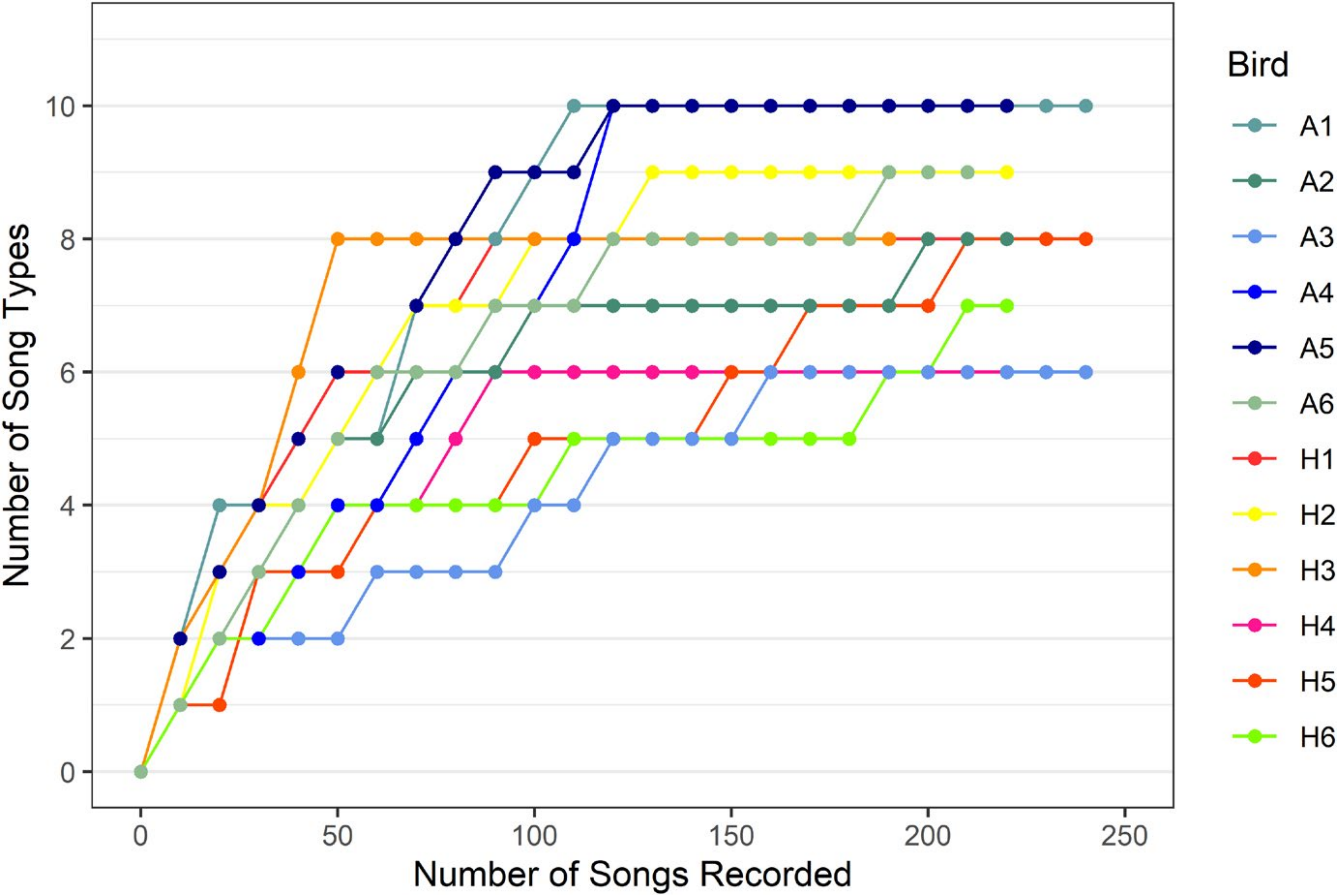
Number of song types recorded as a function of number of song instances recorded for a sub‐set of individuals. Cooler colors represent birds recorded at the agricultural site (‘A’) and warmer colors represent birds recorded at the human‐dominated site (‘H’)

Cassidy (1993) showed that continuously recording 206 Song Sparrow songs, or 280 songs on multiple days, was sufficient to attain a 0.95 probability of acquiring the entire song repertoire in the population that she studied. In another population, Potvin et al. (2015) found that 200 songs, not necessarily continuously recorded, were required on average to acquire a full song repertoire. Similar to Boogert et al. (2011), we chose 200 songs as the threshold to provide a measure of song repertoire size (mean number recorded perindividual: agricultural = 229.1; human‐dominated = 222.9) and reached that threshold for some birds by recording on different days. For two individuals, we recorded a total of 190 songs and 196 songs, for which all new song types had been uttered before 50 and 90 song instances had been recorded, respectively. Effort curves created for a subset of individuals (*N* = 12, with 6 from each site) from our study showed that on average all new song types had occurred before 140 song instances were recorded (Figure 1; range = 50 to 210). We assume that if we underestimated song repertoire sizes, given the similar asymptotes that we observed between sites (Figure 1), then we underestimated equally at both sites and so the comparison between sites is valid.

**FIGURE 2:**
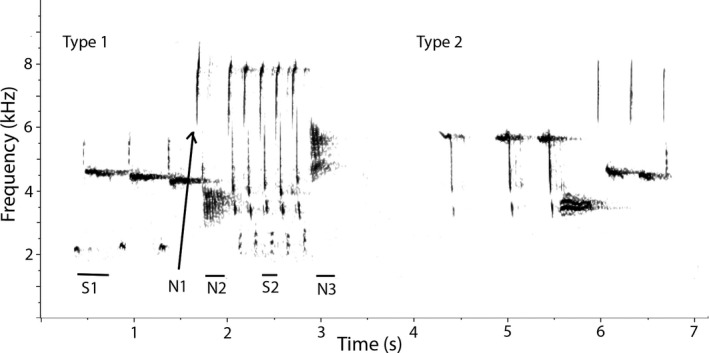
Two different song types. Note that in song type one, there are two different syllable types, as well as three notes not part of a syllable, labelled. Song type one and song type two do not share any individual note or syllable types

Spectrograms (generated in Raven Pro 1.5) were visually analyzed to establish the number of song types in each bird's song repertoire. This general approach is commonly used for Song Sparrows (e.g., Nordby et al., 2002). Because Song Sparrows utter song bouts with eventual variety (e.g., A, A, A, B, B, B, …), it was generally simple to differentiate between song types (Figure 2) based on when an individual switched from displaying one distinct type to another. In rare cases where song type categorization was not obvious, we compared the song instances regarding syllable types and individual note types (‘element types’; Figure 2). If a song instance shared 50% or more element types with another song instance, regardless of order, then those instances always were considered the same type. If <50% of element types were in common, then we classified the compared song instances as different types. When comparing instances with differing numbers of element types, these rules were used to determine if the song instance with less element types should also be considered a distinct type based on its similarity to the other instance. Though the 50% threshold is arbitrary, we chose it because it ensured that instances classified as the same type were as much alike as different regarding element types.

To analyze each bird's songs, we began with Raven Pro's default settings (window type = Hann; FFT window size = 512; overlap = 50%). We measured the following variables: peak frequency (frequency with greatest energy), number of notes (note was considered a continuous trace on the spectrogram), and duration (to determine note rate). Because visual analysis of spectrograms is not appropriate for measuring minimum or maximum frequency in urban environments, due to the possibility of error (Zollinger et al., 2012), we did not measure these variables. In Raven, we began with a brightness of 52 and a contrast of 90 for each song analyzed and adjusted these levels as necessary to make all notes in a given song visible. Brightness was set to the least possible level that allowed for the faintest note in the song to be seen. When rarely necessary, window size was also modified to reveal fine frequency or temporal distinctions between notes. For each bird, mean variable values were calculated after making a single variable measurement for each of the song types in its repertoire. The single song type instance chosen for all variable measurements was the first occurrence on a given recording for which we were sure that all notes were visible (i.e., there was no masking).

### 2.3 Statistical analysis

Because our data violated parametric statistical assumptions, we used two‐tailed Wilcoxon Rank Sum tests (using R v 4.0.2 [R Core Team, 2020]) to compare our agricultural and human‐dominated sites with respect to all variables. We confirmed that song repertoire size, note rate, and peak frequency were not correlated with each other (the highest Pearson's |*r*| was .41). Statistical tests were considered to indicate a ‘significant difference’ if *p* was ≤.05.

### 2.4 Permits

This research was approved by Indiana University Institutional Animal Care and Use Committee protocol # 18–006 and was permitted by the state of Indiana (License # 18–049).

## 3 RESULTS

### 3.1 Habitat measures

**FIGURE 3:**
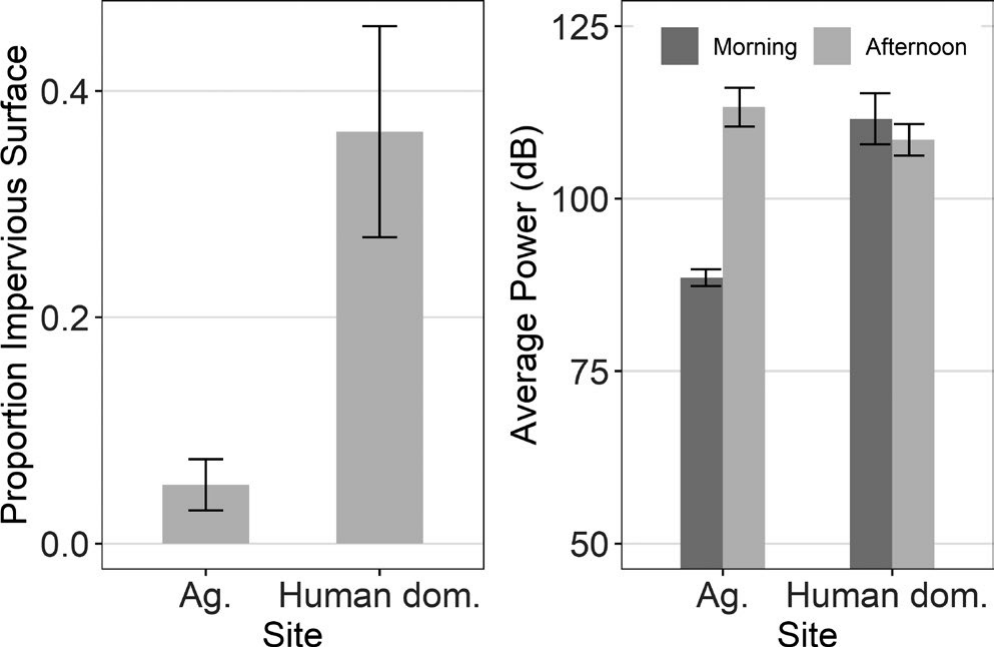
Left plot: Mean impervious surface coverage at five Song Sparrow territories within the human‐dominated site and at five Song Sparrow territories within the agricultural site. Right plot: Mean average power (dB; relative values) at the same territories where impervious surface was measured within the human‐dominated and agricultural sites. Error bars in both plots are one standard error

Noise levels, indicated by relative dB values, were higher at the human‐dominated site than at the agricultural site during the morning session (W = 0; *p* = .01), but not during the afternoon session (W = 17.5; *p* = .35; Figure 3). At the human‐dominated site, there was a greater area of impervious surface (W = 23, *p* = .04) and a mean impervious coverage area that was 600% more than at the agricultural site (Figure 3).

### 3.2 Song complexity and peak frequency

**FIGURE 4:**
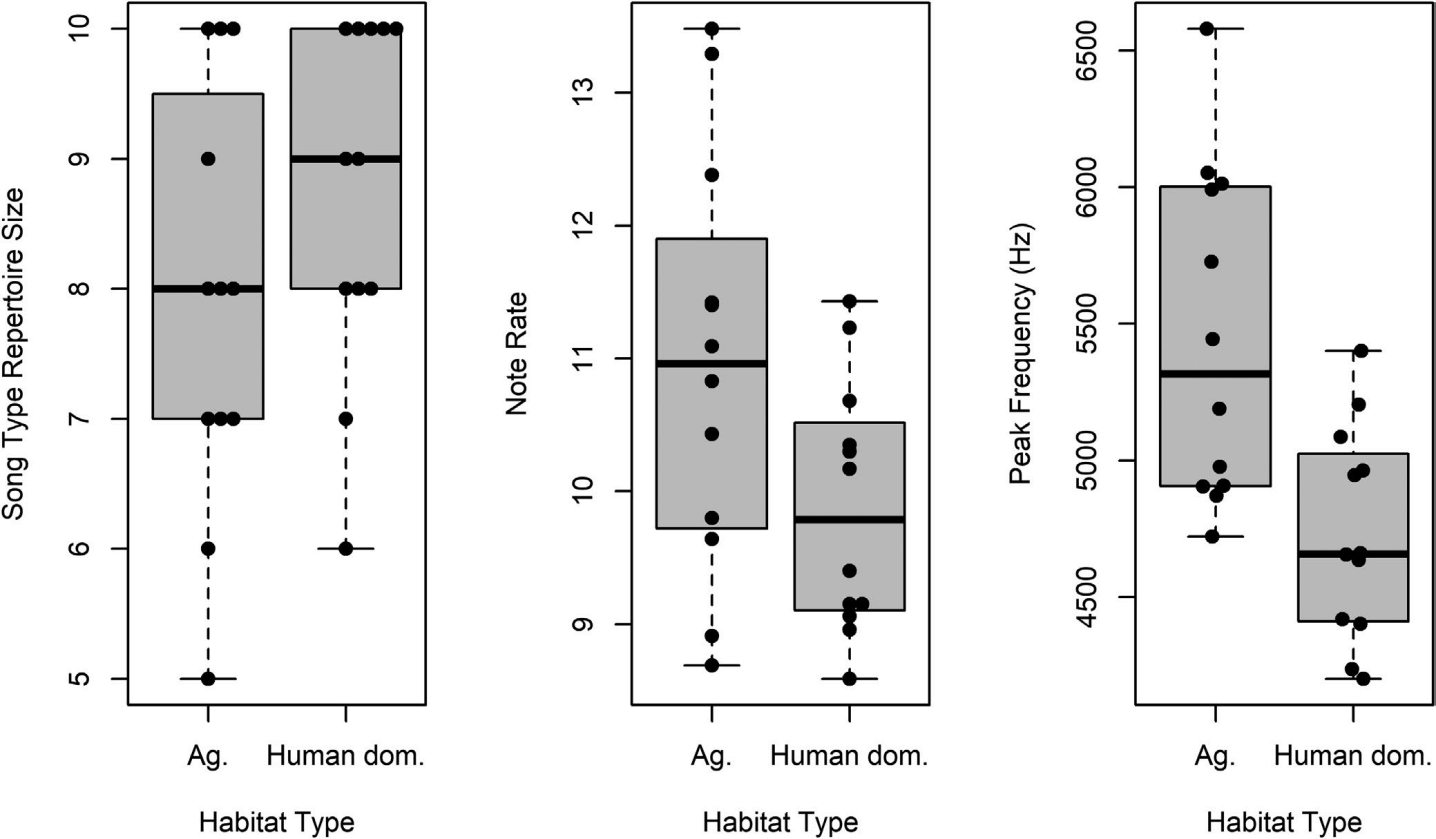
Song repertoire size (plot on left) and note rate (middle plot) did not vary between sites (*p* > .05). Peak frequency did vary (plot on right), with a higher peak frequency at the agricultural site

The median song repertoire size at the agricultural site was 8 (range = 5–10; Figure 4), whereas at the human‐dominated site, the median song repertoire size was 9 (range = 6–10; Figure 4). We did not find evidence that song repertoire size varied between our human‐dominated site and our agricultural site (W = 50, *p* = .20). Note that this *p* value was automatically ‘continuity corrected’ via the ‘wilcox.test’ function in R, to account for ties.

The median note rate within songs at the agricultural site was 11.0 (range = 8.7–13.5; Figure 4), whereas at the human‐dominated site, the median note rate within songs was 9.8 (range = 8.6–11.4; Figure 4). We did not find statistically significant evidence that note rate varied between the sites (W = 101, *p* = .10).

The median peak frequency for songs at the agricultural site was 5316.6 Hz (range = 4720.3–6580.1; Figure 4), whereas at the human‐dominated site, the median peak frequency was 4656.7 Hz (range = 4200–5400; Figure 4). Peak frequency was significantly higher at the agricultural site compared to the human‐dominated site (W = 119, *p* = .01). The Hodges–Lehmann estimator indicated a peak frequency difference of 661.3 Hz (95% confidence interval = 243.7–1209.8).

## 4 DISCUSSION

Our comparison of two sites that varied in degree of human impact (Figure 3) provides further, preliminary evidence about how urbanization, an increasingly common challenge for wildlife (Seto et al., 2011; Shanahan et al., 2013), may affect birdsong (Figure 4). It must be emphasized, however, that all of our results pertain only to the two sites that we studied. Further work must be done to determine if our results generalize to other sites. Nonetheless, particularly the description that we provide of song complexity at an urban and a rural site could be a useful reference for future investigators. Our study is the first that we are aware of to compare song repertoire size based on distinct song types between an urban and rural environment for any species, perhaps because of the time‐intensive nature associated with documenting entire song repertoires for many of the species that sing multiple song types.

Though it has been shown that Song Sparrow populations can vary geographically regarding song repertoire size (Peters et al., 2000), we did not find evidence that song repertoire size was different between our sites (Figure 4). Thus, song repertoire size as a signal, for example, to potential mates (Reid et al., 2004) or to other males (Stoddard et al., 1987), does not appear to have been substantially disrupted by urbanization at our human‐dominated site. Given that total number of syllable types repertoire‐wide (herein: ‘syllable type repertoire size’) has been shown to be strongly correlated with song repertoire size in Song Sparrows (MacDougall‐Shackleton et al., 2009), presumably this measure of song complexity is also the same between our sites. Equivalence in syllable type repertoire size between urban and rural populations of songbird species has been previously demonstrated (e.g., Potvin & Parris, 2012).

Song complexity, via number of syllable types within songs, did not appear to be affected by noise (a salient factor in urban environments) in 11 of 14 Oscine species investigated in one study (Ríos‐Chelén et al., 2012, overviewed by Brumm & Zollinger, 2013). Of the three species on which an effect was detected, at noisier sites two had less syllable types per song and one had more. Hill et al. (2018) did not find differences in number of syllable types per song due to urban factors. Presumably, investigating number of syllable types within entire repertoires, rather than within songs, could have produced different results in these studies. Our study and others may have failed to find a difference in repertoire size due to urban factors because those factors were not sufficiently strong at the site or sites studied. Juárez et al. (2021), for example, showed that ‘element’ repertoire size (measured repertoire‐wide) tended to be negatively correlated with noise at higher levels but not at the lowest levels. Conversely, Deoniziak and Osiejuk (2019) found that thrushes tended to have larger syllable type repertoire sizes in urban compared to rural habitats, which they suggest could be related to higher quality habitat at urban sites. Studies such as these which do find differences in song complexity due to urban factors could also relate condition of young birds to these factors and so link them to nutritional stress. If urbanization is related to nutritional care during development and consequently is also related to a measure of song complexity (e.g., song type or syllable type repertoire size) for a focal species, then that knowledge could be useful from a management perspective. For example, city managers attempting to promote avian health could use such song characteristics as a non‐invasive measure of the success of management actions by recording the songs of species whose song type or syllable type repertoire size is known to correlate with condition.

Note rate, our measure of temporal complexity, also did not vary in a statistically significant fashion at our chosen alpha level (*p* = .05) between our human‐dominated site and our agricultural site (Figure 4). However, given our small sample size, our results provide some evidence that, as we predicted, urban factors at our human‐dominated site may decrease note rate. If an effect exists, it appears to be small (~1 note per s; Figure 4). Results of studies investigating temporal complexity have been mixed. Potvin et al. (2011) found that urban birds sing slower (less temporally complex) songs relative to rural birds based on syllable rate, Hill et al. (2018) similarly found that inter‐syllable intervals at urban sites were longer (i.e., songs were less temporally complex), and Nemeth and Brumm (2009) found no difference between sites based on inter‐element intervals. When it occurs, variation in temporal complexity could have fitness consequences. For example, syllable rate has been associated with female attraction (for broad bandwidth songs; Drăgănoiu et al., 2002). It is possible that more noise at urban sites, and differences in noise reflectance due to ‘canyon effects’ (Warren et al., 2006), could in some cases mask or distort, and therefore diminish the value of songs with greater temporal complexity and so decrease their usefulness for the selection of males by females. Our human‐dominated site largely lacked tall buildings close in proximity and so ‘canyon effects’ likely did not occur often. This may have contributed to weakening the effect on note rate at our human‐dominated site. However, our study did not address such specific causes. Therefore, bird condition and/or care during development—as well as many other factors that we did not measure—could be the primary cause for the possible difference in note rate between our sites. Future investigators should also consider that particular components of songs, such as trills (Redondo et al., 2013), may transmit better in urban environments if elements are uttered at a higher rate. This aspect of Song Sparrow song, however, was beyond the scope of our study.

Peak frequency of songs at our agricultural site was higher compared to our human‐dominated site (Figure 4)—with a relatively large difference in frequency (~661 Hz)—which was contrary to our prediction. As has been commonly found regarding minimum frequency (Seger‐Fullam et al., 2011; Slabbekoorn & Boer‐Visser, 2006), including in Song Sparrows (Wood & Yezerinac, 2006), peak frequency in birdsong has been shown to shift up in noisier areas (Walters et al., 2019) perhaps to avoid signal disruption by low‐frequency noise. However, only nine of 35 studied species overviewed by Brumm and Zollinger (2013) were found to have peak frequency affected by noise (eight studies showed higher peak frequency at noisier sites, one study showed lower peak frequency). The study which showed a lower peak frequency where it was noisier was not conducted in an urban area and the shift appears to have been due to singing at a frequency below insect noise (Kirschel et al., 2009). Because we did not record noise levels at each Song Sparrow territory, our ability to make inferences about the cause of the peak frequency difference in our study is limited. However, future investigators may wish to determine if lower peak frequency at human‐dominated sites compared to agricultural sites occurs in other such pairings of Song Sparrow populations. If our observations are a part of a larger trend for this species, then there could be undiscovered causes for this occurrence beyond ambient noise levels. It is possible that urban noise does not tend to affect peak frequency of Song Sparrow songs because of the tendency of this song characteristic in this species to occur at relatively high frequencies (>4 kHz) that may not be substantially masked by urban noise. Higher peak frequency at the agricultural site could actually correspond with previous studies (e.gBillings, 2018; Nicholls & Goldizen, 2006) which have found that, in accord with the acoustic adaptation hypothesis, birds in more open habitats tend to utilize higher frequencies compared to more closed habitats. For example, Job et al. (2016) found that a sparrow species tended to utter songs with lower peak frequency at sites with more urban structure, which may have helped individuals to avoid signal disruption associated with reverberation.

Future investigators should consider, and perhaps improve upon, several aspects of our study. Identifying subjects as migratory or sedentary, especially in partially migratory species like Song Sparrows, would help to disentangle associations of these strategies from effects of urbanization. Urban individuals, for example, may be more likely to be sedentary (Partecke & Gwinner, 2007), which could affect the way that birds sing (Nelson et al., 1996) independently of factors like urban noise. Another limitation of our study is that we only compared two sites which could feasibly vary due to cultural factors not related to urbanization. Ideally, multiple urban–rural pairings would be compared so that results are less specific to a particular urban–rural pairing, and are therefore more generalizable. Comparing birdsong characteristics, like song repertoire size, between less disturbed rural sites and areas more heavily impacted by urbanization than we used in our study may increase the likelihood of identifying effects due to urban factors. For example, downtowns of large cities or sites in close proximity to busy roads could better serve as urban sites than the university campus setting that we used. Similarly, sites more undisturbed by humans than our agricultural site was, which lack occasional disturbances like noise from farm equipment, may better represent the rural category. Daily patterns of high‐ and low‐frequency noise should also be considered between sites by future investigators. Another improvement upon our study would be to control for the age of birds recorded. If Song Sparrows with larger song repertoire sizes tended to live longer at our study sites, as Hiebert et al. (1989) found, then variable age distributions between sites may have masked effects of urban factors on individual song repertoire sizes. In our study, for example, Song Sparrows of the same age could vary regarding repertoire size, whereas the overall population of singing individuals does not. Categorical designation of our sites as ‘human‐dominated’ and ‘agricultural’ was sufficient to complete our exploratory goal of broadly comparing song complexity and peak frequency between sites. However, a regression approach which incorporates predictor variables such as noise or artificial light at night levels at each Song Sparrow territory would more specifically address possible urban effects on the response variables that we measured. Such microhabitat features are known to affect attributes of birdsong (Fernandez‐Juricic et al., 2004), as does another factor which we did not model, namely bird density (Hamao et al., 2011).

Urban influences on birdsong frequency have apparently been more commonly investigated than have effects on song complexity. Perhaps, as supported by our results, frequency tends to vary more due to urban factors than does song complexity. However, given the importance of song complexity in mate attraction and territory defense, understanding how this aspect of birdsong varies in response to urbanization may help to predict how individual species are being, or will be, affected by urbanization. Additional studies of song complexity across an urbanization gradient will help us to better understand birds in urbanizing environments and could even inform avian conservation efforts. Our pilot study could be especially useful to those interested in designing a full‐scale study meant to establish relationships between song repertoire size and urban factors.

## CONFLICT OF INTEREST

We have no competing interests.

## AUTHOR CONTRIBUTIONS

**Dustin E. Brewer:** Conceptualization (lead); Data curation (lead); Formal analysis (lead); Investigation (lead); Methodology (lead); Project administration (lead); Writing – original draft (lead); Writing – review & editing (equal). **Adam M**. **Fudickar:** Conceptualization (supporting); Funding acquisition (lead); Investigation (supporting); Methodology (supporting); Project administration (supporting); Writing – review & editing (equal).

## Data Availability

All data discussed herein, and code, are available via Dryad (Brewer & Fudickar, 2022) at this link: https://doi.org/10.5061/dryad.h70rxwdkv.
